# Psychology and Spirituality: Reviewing Developments in History, Method and Practice

**DOI:** 10.1007/s10943-022-01731-1

**Published:** 2023-01-11

**Authors:** Sally Sargeant, Jacqui Yoxall

**Affiliations:** 1 Psychology and Health Communications, Southern Cross University Faculty of Health, Bilinga, Gold Coast, QLD 4226 Australia; 2 Chair of Discipline (Allied Health) and Director of Clinical Services, Southern Cross University Faculty of Heath, Lismore, NSW 2480 Australia

**Keywords:** Psychology, Spirituality, Measurement, Communication, Practice, Flow, Psychological capital

## Abstract

This paper begins with an overview of the governing principles of psychology as a discipline, and outlines the key paradigm shifts that potentially aligned with concepts of spirituality from the early twentieth century to contemporary theory. The discussion then moves to consider how research methods in psychology can contribute to understanding how spirituality is measured and described. We consider the contribution of validated surveys, and how qualitative methods can access the lived experience of spiritual phenomena. More specifically, the psychological constructs of "Flow" and properties associated with psychological capital are posited in relation to characteristics that define a spiritual experience. Finally, we draw attention to how exploration how spirituality of individuals may be addressed, and the scope for including spiritual appreciation in competencies required in psychological practice.

## Introduction: Aims and Scope

At the outset, it is important to articulate the aims of this paper. Charting a path between the title components is an attempt to link the parallel, crossing, and indeed divergent paths of spirituality and psychology. While the topics herein are presented as foundations upon which to build a discussion about the relationship between a broad concept and academic discipline, it is not a comprehensive review. For example, while aspects of how spirituality can be measured within the survey methods often deployed in psychology, a full review of questionnaires measuring spirituality is not present. Such an endeavour would warrant a separate paper, as the development, testing, and refining of such measures deserves appropriate attention that extends beyond the scope of this discussion. Furthermore, spirituality (as opposed to religion) is discussed with relation to psychology here. The specific reason for this is due to spirituality generally being a broader concept that embraces individual approaches to the pursuit what is sacred. Religion is generally characterised by a more prescriptive approach to pursuing the sacred (Hill et al., 2000), which to a large extent counters the tenets of individual differences that fortify routes to psychological well-being. It is for this reason that we champion spirituality as the concept to explore in relation to psychology.

## Historically Positioning Spirituality within the Discipline of Psychology

As a discipline, psychology traverses the arts and sciences. Our behaviours, emotions, and cognitions are inextricably linked to everything we experience in our lives, from the ordinary to the extraordinary. To unpack the relationship between psychology and spirituality, it is necessary to explore how the discipline of psychology emerged from a wealth of philosophical questions to the standards of scientific inquiry it upholds today. It was towards the end of the nineteenth century that Wilhelm Wundt, described as often the “Father of Psychology” (Burton et al., 2018), championed mental processes and the integral positioning of the mind in human existence:

From the standpoint of observation, then, we must regard it as a highly probable hypothesis that the beginnings of the mental life date from as far back as the beginnings of life at large*.* (Wundt, 1904, 31.)

Wundt established the first psychological laboratory in Leipzig, Germany, not deeming experiments as the only routes of enquiry to determine psychological knowledge and promoting the necessity to study elements of the mind. This flexibility was, however, short-lived. Wundt’s student, Edward Titchener, argued that the mind was instead comprised of components and that if these components could be defined and understood then the structural processes of the mind could be explored. He used introspection as a tool to examine the component of consciousness. This generated the rise in experimental psychology and became known as “structuralism” (Titchener, 1902).

Another school of thought simultaneously emerged. “Functionalism” asserted that psychological processes were determined by individuals’ functioning in, and adaptation to their environment. In 1890, William James had proposed that there was little utility in building a typology of consciousness, and instead asserted that explanation rather than description warranted greater attention (Hart, 1981). Structuralism and functionalism were the key platforms upon which psychology began to exhibit its academic presentation. The latter framework leant itself to incorporating spirituality as a potential component through which to understand and explain consciousness. For example, if someone engaged in spiritual practice, his or her adaptation to their environment could partially be explained. This association was not, however, made apparent at that time.

Psychological inquiry then became ensconced within what is perhaps the most well-known paradigm of psychodynamic theory. With Sigmund Freud at the helm of psychoanalysis, emphasis partially shifted from the study of conscious awareness to the argument that powerful unconscious motives determined our actions (Holmes, 2017). Freudian concepts, such as denial and repression, are still discussed in popular psychology today, despite the difficulties in testing such theories with any degree of scientific rigour.

Scientific objectivity and experimentation gathered momentum through another major psychological approach—behaviourism. The core premise of behaviourism is that only measurable aspects of behaviour should be considered and that internal events are neither relevant nor necessary to understand in order to effect behaviour change. The main assumptions within this approach focused on learning that was not influenced by internal or inherited factors, but was, rather, specifically in response to the environment. Ivan Pavlov is the most well-known proponent of behaviourism. His studies about the digestion of dogs, and his work in training dogs to salivate at the sound of a bell still warrant inclusion in contemporary psychology teaching programs (Akpan, 2020). Pavlov introduced an important descriptor relating to behavioural response—conditioning. The pairing of an experience with a stimulus produces an ongoing association between the stimulus and the physiological response to that experience. John B. Watson’s work was a powerful demonstration of the influence of frightening experiences on learning and behaviour, and the maintenance of behavioural responses to traumatic events into the longer term (Watson, 1913). Our understanding of aspects of human response to trauma today—particularly the concept of “triggers” to traumatic memories and associated physiological arousal—are based upon these theories. In proposing that learning and subsequent behaviour occurs as a result of punishment or reward, B.F. Skinner (1953) progressed behaviourism further into the twentieth century. The shaping of human behaviour using operant conditioning methods continues today to be the basis of most forms of animal training and human behaviour modification strategies. However, absolute disregard of internal experiences, including emotions, beliefs, and attitudes, was very difficult for some psychology scholars to accept. So too was Skinner’s ultimate premise that free will does not actually exist and that all behaviour, human and animal, is simply determined by environment.

Although behaviourism promoted experimentation, objectivity, and further legitimised psychology as a science, there was little room for considering introspection and the subjective experience of what it was to be human. The deterministic stance of behaviourism began to be questioned as theorists championed individual needs and aspirations as essential parts of psychological inquiry. Ultimately Skinner’s work was so focused on observable and measurable behaviours that the “Skinnerian Era” promoted a counter-reaction from those who maintained that the internal, “unseen” workings of one’s inner world is a core component of the human experience. One of the most well-known proponents of this counter-reaction was Abraham Maslow.

Maslow (1943) proposed that all human beings have an inherent motivation to develop and grow towards better existence. Influenced by the atrocities of the Second World War, Maslow argued that before psychological or spiritual growth can occur, a person must have the basic requirements of daily life—shelter and food. The hierarchy of needs detailed specific human needs in a model of overlapping stages, including basic needs, safety needs, social needs, self-esteem and ultimately concluding at a stage he termed, “self-actualisation”—becoming the most that one can be. Even though the hierarchy of needs was more of a concept than a working model that could be operationalised and measured, the idea of such an inherent drive to seek a better-self continues to be cited across clinical, professional, and educational psychology domains today, also cementing a firm place in popular psychology.

When examining the role of spirituality in psychology, Maslow’s theories could be considered as the early contemporary return to considering the spiritual mind within the human experience. The concept of self-actualisation and what this may encompass for different individuals has been extensively debated in scientific literature and utilised widely in therapeutic work, including responses to chronic disease and illness (Bayoumi, 2012), palliative care (Zalenski & Raspa, 2006), posttraumatic stress (Lonn & Dantzler, 2017), and addiction (Best et al., 2008), and offending behaviour and rehabilitation. In his later years, Maslow expanded upon his proposed hierarchy by adding the stage of Transcendence. Transcendence was conceptualised as the stage beyond being the best version of oneself that a person can be, to something beyond the self, for the benefit of others or the community or world—that is, altruism or spirituality. The desire to reach this level of development, that one’s existence and meaning involves a level of spirituality is proposed to be the highest level of human consciousness (Koltko-Rivera, 2006).

Although criticised for a lack of scientific evidence to support his theories, Maslow’s work formed the early stages of a new psychological approach, known as Humanism, which was pioneered by Carl Rogers (Rogers, 1951, 1959). Humanism rejected the tenets of behaviourism and augmented Maslow’s work. When appraising the progression of conceptual issues in psychology, this movement was by far the closest connection to aspects of spirituality—simply by acknowledging the variations in individual needs to self-actualise, the door was open for spiritual needs to be embedded within the tiered stages of Maslow’s hierarchy of needs, and clearly aligned to the later addition of Transcendence to that hierarchy.

The Rogerian approach to understanding aspects the underpinning principles of the “therapeutic relationship” and person-centred counselling practiced by psychologists and various other health practitioners to the current day (Rogers, 1951). In practice, the Rogerian therapeutic relationship created a safe space of unconditional positive regard, within which personal insights could be realised and psychological/emotional growth could occur. The concept of unconditional positive regard could be argued to be akin to aspects of a spiritual perspective or approach to relationships with others—that is, the conscious decision to set aside any biases, emotions, attitudes, or beliefs in order to generate and offer positive regard to another person without any conditions or expectations attached.

The oscillations of psychology between philosophical and scientific inquiry veered closely towards the spiritual, particularly during the 1960s when the Humanism movement was at its strongest. The fundamental question was whether human behaviour was passive and controlled (and therefore determined) by the environment within which a person existed, or whether internal experiences (emotions, beliefs, intentions, values, etc.) should be considered to best understand how we experience, and interpret, reality.

Psychology as a discipline advanced through the twentieth century, with the affiliation to the observable, the concrete, and the elevation of experimental methods as agents in psychological research remaining in focus. Earlier theories of behaviour expanded to accommodate the influence of human thought, attitudes, beliefs, and emotions, which could be tested to some degree by self-reporting of such thoughts. As before, psychological theories that were most amenable to scientific examination provided an evidence base for interventions such as cognitive behavioural therapy and as a result this area of thought and work flourished. This left limited space for the consideration of spiritualism and religiosity, and a clear division between behaviour, thought, emotion, and the soul. Although the Positive Psychology movement of the 1990s, spearheaded by Martin Seligman (Seligman & Csikszentmihalyi, 2000), essentially brought about a contemporary wave of Humanism, and argued for a focus on psychological well-being rather than psychopathology, support for it remained generally in popular psychology because of the same challenges with measurement and observation that had plagued this area of enquiry in the 1950s. The advancement of the rigorous application of the hypothetical deductive method added to the viewing of psychology as a science, which effectively consigned spirituality and religion to clergy and related professions, while psychologists directly addressed behaviour.

## Measuring and Defining Spirituality in a Psychological Context

It is useful at this juncture to consider the alignment between methodological paradigms with psychology and spirituality. Psychology developed in a tradition of quantitative research within the scientific method, which specifically sought to define and measure identified variables relevant to the research question. Although qualitative paradigms were acknowledged, this form of enquiry did not command attention in psychology until the latter half of the twentieth century. By the 1980s, psychologists who had studied largely in the realm of quantitative methods championed the need for more qualitative approaches to become more visible. More specifically, they advocated for the same rigour and quality standards to be applied to such methods.

Qualitative methods generate in-depth data and therefore provide scope for exploration of particular phenomena instead of explanation. The increased use of lengthy interviews, focus groups, diary methods, and photo-elicitations has vastly expanded the ability of psychology researchers to pursue subjective experience, and investigate what experiences mean to individuals through consideration of data collection methods. Table 1 provides a basic comparison of the philosophical underpinnings of positivist and phenomenological paradigms that govern these methodological frameworks. These methodologies lend themselves to explorations of spirituality in human experience, framed within psychological context. An enhancing component of qualitative methods is the requirement to demonstrate quality checks in the deployment of these methods through processes of reflection and reflexivity (Willig, 2019). Rather than simply directing research questions to subjects /study participants, researchers and psychologists are required to consider how their own actions, questions, assumptions, and beliefs affect the course of inquiry, thereby leaving sufficient room for spiritual experiences and beliefs to be explored and accounted for, depending on the research question and context.

**Table 1:** Characteristics of positivist and phenomenological inquiry

Paradigm	Positivism	Phenomenology (non-positivist)
Characteristics	Seeks to explain experiences	Seeks understanding of experiences
	Reality comprises indisputable facts	Reality is complex with many factors
	Causal links make sense of the world	One makes sense of one’s own world
	Knowledge is experienced	Knowledge is acquired

So far, this commentary has centred on the scope of psychology to include aspects of spirituality though its theoretical developments and methodological boundaries. It has addressed issues of measurement and experimentation, along with the broader philosophical paradigms aligned to psychology, and how spirituality might be positioned within these schools of thought. However, another possibility arises that potentially bridges these different paradigms, which is the question of how to measure aspects of spirituality. To date, this challenge has been approached both from a quantitative and qualitative perspective.

A key non-experimental method that forms the backbone of much research in social psychology is the self-report survey. Surveys have been developed in many forms, from open-ended questions to closed statements that force the respondent to pick from a limited number of predetermined responses. Although this method can still be influenced by researcher bias, there are many examples of reliable surveys used in psychology and other disciplines that have multiple uses, ranging from informing medical diagnoses (e.g. Beck et al., 1961), to identifying personality types (e.g. McCrae & Costa, 1987). Certain behaviours and moods that are well documented and argued to be universally agreed upon, at least in regard to the American Psychiatric Association (2013) or World Health Organisation (2016), are easily inserted into surveys measuring psychological constructs. However, measurement of a construct *without* such a delineated set of observable characteristics, such as spirituality, is infinitely more complex. Spirituality is difficult to define, as noted by Chiu et al. (2004), as it means different things to different individuals, groups, and cultures. It is reasonable then to question whether spirituality can be “measured”.

As spirituality has become more widely recognised as a component of healthcare worthy of attention, there is growing evidence attesting to the merits of measuring spirituality and documenting its relevance to clinical settings. However, spirituality is not considered a univariate construct to measure. Established questionnaires within this subject area often address a particular aspect of spirituality, such as “spiritual well-being” or “spiritual coping”. A systematic review by Monod and colleagues (2011) examined 35 questionnaires that measured spirituality in clinical research and proposed a typology of instruments to reflect the multivariate nature of this complex area.

The earliest one identified emerged in 1983; the Spiritual Well-Being Scale (SWBS). The SWBS was developed in response to a perceived lack of recognition for the significance of religion and existentialism in assessing quality of life in connection with well-being. The questionnaire comprised of 20 items and was found to be reliable on test–retest bases. A later addition to measurement scales in this domain was the Spirituality Index of Well-being, a 12-item scale comprising statements relating to self-efficacy and life schema (e.g. I haven’t found my life’s purpose yet). It was found to correlate more strongly with established measures of well-being rather than the SWBS (Frey et al., 2005), thereby signalling a degree of utility within health psychology research.

Other examples of self-report surveys and measures fall within the dimension of spiritual coping. The Spiritual Beliefs Inventory (SBI-15) is a 15-item questionnaire designed to assess religious and existential beliefs and has been used within a quality-of-life framework for assessing adjustment to illness (Holland et al., 1998). Other measures were developed to examine the role of spiritual coping in general areas and in response to specific events, including traumatic events. A notable example is the use of the Spiritual Support Scale, in a study exploring the role of spiritual coping following events of the 11 September 2001, terrorist attacks (Ai et al., 2005). Understanding human response to trauma is important to psychologists, not only in regard to the experience of grief, loss, shock, and sometimes psychopathology. Posttraumatic growth and personal and spiritual development have also been observed to be a response to trauma for some people, which resonates with the aforementioned tenets championed by Maslow, Rogers, and Seligman.

## Suggested Psychological Frameworks of Spiritual Experiences

Unlike strong negative emotional reactions generated by worldwide events, some every day experiences can generate feelings of well-being and spiritual connectedness for some people. Research indicates that distinctly productive activities and situations can generate a feeling of spiritual well-being, with the sense that everything runs smoothly and without complication. Many people may describe an intense period of productivity or concentration as being “in the zone”. The concept of “Flow” was popularised by Mihaly Csikszentmihalyi (1990), largely though his seminal work on human optimal experience. Within this work, he described Flow as a meaningful state that made life worth living, stating that “you know what up need to do is possible to do, even though difficult, and sense of time disappears. You forget yourself. You feel part of something bigger” (Csikszentmihalyi, 1990, as cited in Moore, 2020). Earlier in this paper a definition of spirituality observed a search for the “sacred”, a clear indication of something possibly intangible and certainly greater that oneself. Csikszentmihalyi also asserted that Flow was characterised by a loss of self-consciousness—implying that the perception that one’s actions and performance are driven by greater influences, and thereby potentially aligning these influences within a spiritual as well as a psychological domain.

Flow has been examined in many contexts, including sport, creativity, and workplaces. Within sports psychology and research into athletic performance, Flow in connection with spirituality is identified as a key facilitator of peak execution (Watson & Nesti, 2005). While measurable performance excellence is observed by quantifiable outcomes in competitive sport, holistic, and personal well-being are other factors that enable an individual to flourish. In another context relating to emotional experiences of performing music and or choral singing, the search for meaning in performance was aligned with experiencing Flow. In a qualitative study, Lamont (2012) found that individuals reported positive emotions as a result of individual and group performance. However, in Lamont’s study, participants did not describe “losing themselves” in the sense that Csikszentmihalyi originally theorised, suggesting that the Flow state may not always be characterised by this. Lamont surmised that this may be due to priming and practicing music that is not necessarily chosen by those performing, but simultaneously argues that the Flow state still occurs and may be aligned to spiritual experiences.

These explorations of Flow in sporting and musical contexts lead to other considerations of how psychological factors linked to spirituality manifest in individuals and in groups. Social Identity Theory (Tajfel & Turner, 1986) posits that we behave differently in groups to when we are alone. Empirical research on Flow, and emotional experiences in spirituality affirms this to a degree, in a comparison of experiences between people attending a Catholic mass (group), individuals engaging in Zen meditation, and a control group (Rufi et al., 2016). Those attending the mass returned higher scores and statistical significance in five out of the nine dimensions on the Flow State Scale—these being ability level (challenge), clear goal, control, action-awareness merging, and concentration (Jackson & Marsh, 1996). Although this is insufficient to imply certainty, it demands further attention to the implications of spiritual experiences being attained more easily in group rather than individual contests.

While we can locate examples in certain social and environmental contexts within psychological research, it is harder to find evidence of spirituality specifically in connection with psychological therapeutic practice. However, the tenets of fulfilment, optimisation, and Flow in connection to spirituality all fall within the positive psychology movement and the work of Martin Seligman. Positive psychology has brought us closest to the exploration of one’s spiritual self as part of work towards establishing psychological well-being within a meaningful and satisfying “good life”. As referenced earlier, the emergence of positive psychology in the 1990s was somewhat of a return to some of the humanistic theories of the 1960s. Within the positive psychology field, spirituality is considered as an aspect of the human experience, and often as a tool to enhance resilience in the face of adversity, and as a compass for navigation of a fulfilled life. Positive psychology has allowed public, non-academic access to the psychology discipline in a greater capacity than before. The rise of popular psychology focusing on mindfulness, meditation, acceptance, and other strategies (adopted from Buddhism and other belief systems) is now scattered throughout the grey psychological literature. However, the scientific evidence base to some of this work is still be established.

Seligman’s work on the enhancement of the human experience rather than a focus on dysfunction captured a wider popular audience of the potential contribution of psychology to the daily lives that people lead. Positive psychology and the concept of Flow gathered momentum towards the end of the 1990s, and by 2000, advances occurred in the empirical and theoretical development of promoting optimal experience and emotional health. Seligman advanced the work of Csikszentmihalyi, acknowledging the loss of self-consciousness as a result of Flow and exploring the how kindness and or altruism mediated the Flow state. This program of work yielded classification of six virtues—wisdom, courage, humanity, justice, temperance, and transcendence—effectively a typology of ideals to lead an optimal life within a positive psychology framework (Peterson & Seligman, 2004). Following a cross cultural study of religious texts, “spirituality and transcendence” was a clearly identified virtue, attainable through cultivation of signature strengths and an apparent declaration of the alignment of spiritualty to psychological well-being. The emphasis on strengths and virtues mirrors the concept of having psychological reserves within which to determine the links between the psychological and the spiritual, and leads to another term that has recently entered the domain of positive psychology.

“Psychological capital” (or Psycap, a shortened version) largely emerged within a business/corporate context and is often referred to in leadership focussed literature. It is, however, also a concept that overtly acknowledges spirituality as a potential contributory factor in attaining high psychological capital. Psycap is largely defined according to four criteria deemed to determine an individual’s development of a positive psychological state through (1) necessary confidence and efficacy to address challenging tasks, (2) sustaining optimism through to completion of said tasks, (3) persevering and, if necessary, changing plans to attain various goals, and 4) deploying resilience in adversity (Luthans et al., 2015). When assessing characteristics of spirituality alongside those aligned to Psycap, there are clear parallels which affirm the former as a potentially major contributor to Psycap. An example is those of a spiritual leaning being able to cope with hardships—within and outside of occupational contexts (Pargament, 1997). The relationship of Psycap to Flow is also important. As discussed earlier, Flow is characterised by distortion of time and loss of self in task completion. Psycap also introduces authenticity of self as a positive component; authenticity in itself as historically been described as an “absence of self-deception” and being true to oneself (Sartre, 1966), which further connects the characteristics that complement spirituality and resonate with the Rogerian ideals of Humanism.

Flow, Psycap, authenticity and the general popularity associated with positive psychology lends a theoretical hand to understanding the intersection of psychology and spirituality. However, the application of spiritual content within psychological practice is somewhat hard to identify. At this point, it is useful to examine the presence (or absence) of spirituality in competencies required to become a practicing psychologist or engage in clinical supervision.

## Spirituality in Psychological Practice and Competencies

Although paradigm shifts in the history of psychology do not directly speak to a relationship with spirituality, it is fair to say this relationship is more than just a flirtation. Psychologists have been involved in the assessment and therapeutic treatment of individuals since the aftermath of the Second World War. Interest in personality research grew after the atrocities of the conflict and also in rehabilitation of wounded soldiers adjusting to civilian life. People began to question how affected individuals coped with trauma associated with physical and sociological disruption. Specifically, the growth of social psychology stemmed from the incomprehension of cruelty that human beings inflicted upon others as evidenced by the Holocaust and the persecution of Jewish people (Byford & Tileagă, 2014).

This upsurge in interest of social explanation and adaptation in the most extreme of circumstances led to the dominance of clinical and counselling psychology, which was concerned with assessment and treatment of clinical symptoms and conditions, or generally the easing of psychological angst and pain associated with the experience of being human (Bazar, 2015). Equally, as noted earlier, it was during this time that a strong dominance of cognitive behavioural therapy (CBT) emerged due to its inherent qualities of measurability and acceptance of being “evidence based”, whereas more existential or humanistic approaches to counselling and intervention were less measurable and quantifiable (Ruggiero et al., 2018). Cognitive Behavioural Therapy remains one of the most recognised frameworks in the application of psychology today.

The influence of positive psychology, and particularly Seligman’s strength-based and virtues framework, is clearly visible in practice approaches and popular culture. Daily expressions of gratitude are encouraged, as are counting acts of kindness and visualising one’s best possible self. (Vella-Broderick, 2011). Empirical studies have also shown how positive psychology interventions have successfully alleviated depression and mood disorders, and have progressed from correlation studies to evidencing this association through meta-analytic approaches (Santos et al., 2013; Varghese et al., 2021).

The evidence, therefore, of positive psychology and associated spiritual domains adding value to the discipline on a theoretical basis and assisting individuals though psychological intervention is considerable. Yet, these associations are not widely broadcasted or explicitly advocated in competency frameworks or standards within educational accreditation bodies and practice regulators. Despite more recent meta-analytic work that has addressed patients’ perspectives on religion and spiritualist in psychotherapeutic practice (Captari et al., 2018), there remains a shortage of worldwide scientific literature that links spirituality to psychology or spiritual care to the practice of clinical, forensic, or other applied areas of the discipline. In Australia, psychology discipline competencies identified by the Australian Health Practitioner Regulation Agency (AHPRA) and the Psychology Board of Australia do not directly refer to spiritual or pastoral care, but acknowledge other constructs related to this within as general health and well-being—not unlike the aforementioned Psycap principles.

Pre-registration Psychology education is also primarily focused on objective inquiry and research methodology, rather than placing emphasis on subjectivity and thereby departing from the phenomenological principles designed to access lived experience (Australian Psychology Accreditation Council, 2019). Instead, competencies mandated within accrediting bodies or associations that potentially address the scope of spirituality are to be found in the post training, wherein certain practice specialities (e.g. counselling, community) are aligned to examining the presence of spirituality in assessment and treatment. Interestingly, some more direct work centres on competencies outside of regulatory bodies in the form of measuring spiritual competence in therapeutic circumstances. North American research recently attended to this domain acknowledging the historical absences of spirituality being documented within formal competency frameworks. While aforementioned scales measured spirituality as a construct, more detailed work proposed specific attitudinal, knowledge-based and skill-based competencies is evident (Vieten et al., 2013). Of particular significance was the recommendation that psychologists can differentiate between spirituality and religion as constructs, alongside helping clients explore their spiritual background and addressing related problems if necessary.

Calls to address such needs are gathering momentum, stating that that failure to do so places psychologists at risk of unintentional failure in building therapeutic alliances (Vieten & Lukoff, 2022). This suggests a positive direction in bringing spirituality more overtly into psychotherapeutic practice, with empirical studies also reporting that clients are increasingly open to discussing religious/spiritual influences on their well-being (Terepka & Hatfield, 2020); however, there remains a lack of distinction between religiosity and spirituality, in such works which may confound successful integration.

Identifying specific problems brings a slightly divergent angle to this commentary, which has so far couched spirituality within some related concepts such as Flow, Psycap, and authenticity in a largely positive light. The competencies proposed by Vieten and Lukoff (2022) encourage recognition of psychological problems that may arise from traumatic experiences relating to organised religion, suggesting the spiritual abuse can and must not be discounted within any competency framework. Spiritual abuse has been defined as:

“coercion and control of one individual by another [..] manipulation and exploitation, enforced accountability, censorship and decision making, requirements for secrecy, pressure to conform, misuse of scripture or the pulpit to control behaviour, [..] the suggestion that the abuser has a “divine” position, isolation from others, especially those external to the abusive context” (Oakley & Kinmond, 2013. 25).

Further work proposing safeguarding policy within the UK also identified key areas aligned to manipulation, shame, and blame as a consequence of spiritual abuse (Oakley & Kinmond, 2014). While not declaring a range of competencies for psychologists and counsellors in this context, the authors make clear links between these themes and the potential of psychology to make valuable contributions to assisting those affected, and educating key stakeholders such as spiritual leaders and educators to recognise how maladaptive behaviour or mental ill-health manifests as a result of spiritual abuse.

Whether supporting clients to live an optimal life by exploring aspects of spirituality, or being alert to signs of harmful consequences of spiritual abuse, it is clear that despite the lack of mandated competencies there is ample scope for psychology to play a critical part in any care relating to this area. Numerous attempts to evaluate religious and spiritual competence in health professions reveal counselling to be the most visible discipline aligned to competence measurement and development, yet psychology as an overarching science has not capitalised on this link in the same way. As outlined earlier, the reluctance to move away from psychology as a “hard” science may account for this—a philosophical standpoint that has not escaped recent appraisal in documenting possible barriers to competency development (Vieten et al., 2013). Another notable barrier concerns psychologists’ possible uncertainty about their role or scope of practice in addressing spiritual concerns, which may hinder or restrict opportunities to establish and maintain a therapeutic alliance (Mrdjenovich et al., 2012). Whatever the barriers or enablers, it is clear that opportunities exist to develop and formalise competencies or safeguarding policies amongst psychological associations worldwide.

## General Discussion

Spirituality is an expansive subject with many definitions. However, it is always concerned with the “inner world” and “inner experience” of humankind, and a set of beliefs that guide one’s interpretation of the meaning and purpose of one’s own life, that of others, and the connection to greater things beyond the human experience. It is, therefore, pertinent to see where connections between spirituality and the discipline of (and practice within) psychology lie. To do so necessitates an appraisal of critical developments throughout the history of the discipline, in which certain schools of thought were aligned to or disconnected from anything concerning the spiritual. Paradigm shifts also generate further thought about methods and methodological principles within psychological science, specifically about aspects of measurement and if/how this can be achieved with singular or multiple dimensions of spirituality.

### Dimensions of Spirituality

When considering specific dimensions of spirituality (e.g. the measurement of spiritual coping) it is reasonable to argue that this advances knowledge of how a traumatic event can lead to an uplifting experience, especially when a psychological response includes a connection to spiritual experiences that are difficult to define and measure. However, specific developments in psychology that conceptually align alongside spiritual dimensions present potential explanatory models as to how these dimensions transpire. Flow and Psycap are highlighted as examples, both of which materialised in the last 50 years, but it is important to acknowledge that such developments should not be considered in isolation as determinants of direct links between psychology and spirituality. Such research and development is unlikely to occur without larger disciplinary paradigm shifts that shape methodological, theoretical, and conceptual frameworks.

### An Evolving Relationship

Other health disciplines have started to explore the role of spirituality in health and well-being, and also in regard to psychological dysfunction and disorder, psychology has to some degree, remained a cautious observer. It is only in the last two decades or so that spirituality has been more carefully considered, most commonly within social psychology and the exploration of personal beliefs, attitudes, and values, and the impact of the same on behaviour. Psychology and spirituality are evidently aligned at a therapeutic level, yet it is surprising how spiritual constructs are not overtly acknowledged in training contexts or therapeutic practice. While these connections may not always be evident, there is balance in the attempts to measure spirituality and Flow within the scientific framework. The associations identified between psychology and spirituality can inform how spiritual enquiry can assist us in promoting psychological well-being, by acknowledging the history of psychology through to current paradigms.

## Conclusion

When trying to articulate how psychological enquiry can help us to understand how and when we embrace what might be spiritual encounters in our lives it is important, if not necessary, to consider historical and current developments in the discipline. Psychologists are called upon to address subjective reality in various realms; however, the dominant historical narrative positioning it as a science does not prepare us well for spiritual care. Understanding oneself and critically enquiring into one’s own biases and attitudes is not part of culture of work, training, or mentoring. Thankfully, the more recent focus on positive psychology—personal growth, development, attainment, and a focus on strength-based approaches—contributes to the discussion of meaning and subjective reality. Psychologists are becoming increasingly involved in all aspects of healthcare, including, but not limited to—rehabilitation, adaptation and response to injury and illness; pain management; aged care; palliative care; oncology; mental health and illness; and human response to trauma. As a discipline, psychology has the power to address all aspects of human adaptation, behaviour, and growth. Given this potential, the inclusion of spirituality alongside psychology in the search for explanatory models of who we are, and what we can achieve, could surely be deemed a positive addition. Human search for meaning is integral to this.
